# Non-Classical Correlations in *n*-Cycle Setting

**DOI:** 10.3390/e21020134

**Published:** 2019-02-01

**Authors:** Kishor Bharti, Maharshi Ray, Leong-Chuan Kwek

**Affiliations:** 1 Centre for Quantum Technologies, National University of Singapore, 3 Science Drive 2, Singapore 117543, Singapore; 2 MajuLab, CNRS-UNS-NUS-NTU International Joint Research Unit, Singapore UMI 3654, Singapore; 3 National Institute of Education, Nanyang Technological University, Singapore 637616, Singapore

**Keywords:** KS Box, PR Box, Non-contextuality inequality

## Abstract

Quantum communication and quantum computation form the two crucial facets of quantum information theory. While entanglement and its manifestation as Bell non-locality have been proved to be vital for communication tasks, contextuality (a generalisation of Bell non-locality) has shown to be the crucial resource behind various models of quantum computation. The practical and fundamental aspects of these non-classical resources are still poorly understood despite decades of research. We explore non-classical correlations exhibited by some of these quantum as well as super-quantum resources in the *n*-cycle setting. In particular, we focus on correlations manifested by Kochen–Specker–Klyachko box (KS box), scenarios involving *n*-cycle non-contextuality inequalities and Popescu–Rohlrich boxes (PR box). We provide the criteria for optimal classical simulation of a KS box of arbitrary *n* dimension. The non-contextuality inequalities are analysed for *n*-cycle setting, and the condition for the quantum violation for odd as well as even *n*-cycle is discussed. We offer a simple extension of even cycle non-contextuality inequalities to the phase space case. Furthermore, we simulate a generalised PR box using KS box and provide some interesting insights. Towards the end, we discuss a few possible interesting open problems for future research. Our work connects generalised PR boxes, arbitrary dimensional KS boxes, and *n*-cycle non-contextuality inequalities and thus provides the pathway for the study of these contextual and nonlocal resources at their junction.

## 1. Introduction

The quantum mechanical description of nature is incompatible with any local hidden variable theory and consequently is said to exhibit Bell non-locality [1]. This counter-intuitive phenomenon finds applications in various quantum information processing tasks such as randomness certification [2], self-testing [3,4,5,6] and distributed-computing [7]. The Bell non-locality can be thought of as a particular case of another under-appreciated phenomenon, referred to as contextuality [8,9,10]. Recently, contextuality has been shown to be useful for quantum cryptography [11,12], self-testing [13] and various models of quantum computing [14,15]. These non-classical correlations are not only present in quantum theory, but post-quantum theories as well [9,16]. It is still not clear if the quantum theory is the only physical theory despite decades of research which makes it pertinent to understand these resources for not only quantum theory but also post-quantum theories, for fundamental as well as practical manifestations [9,17]. In this light, we study some of the relatively less explored nonlocal and contextual resources and discuss possible inter-connections among themselves.

Our focus revolves around the correlations manifested by three different objects from quantum and post-quantum theories with underlying structure governed by the *n*-cycle graph. In particular, we explore the correlations manifested by Kochen–Specker–Klyachko box (KS box), Popescu–Rohlrich boxes (PR box) and scenarios involving *n*-cycle non-contextuality inequalities. The KS box was first introduced by Bub et al. in 2009 [18] who analysed it for a five-dimensional case. The box has a tunable parameter (denoted by *p*), which determines the nature of box namely classical, quantum and post-quantum. Bub et al. showed that it is impossible to simulate the KS box statistics for $p=\frac{1}{3}$ using any classical strategy. For the aforementioned value of *p*, the best classical strategy has a success probability of approximately 0.94667 [18]. The authors showed that the KS box is sturdy enough to simulate the famous PR box—the most nonlocal no-signalling box for the simplest Bell non-locality scenario [16]. It is important to note that PR box has played a crucial role in the understanding of concepts from communication complexity and provide the primary test bed to check against the physical principles to single out quantum theory, which further demands a careful study of these no-signalling nonlocal boxes [17,19,20]. For $p=\frac{1}{2}$, KS box efficiently simulates the PR box [18].

Now, we turn to the last object of our study. The Bell nonlocal nature of theories can be witnessed via the violation of certain inequalities, referred to as Bell inequalities and non-contextuality inequalities in the general case of contextuality [9]. In their seminal paper, Cabello, Severini and Winter showed that certain graph-theoretic numbers give the bounds on these inequalities for classical, quantum and more general theories, namely independence number, Lovász theta number and fractional packing number, respectively [8]. Using the tools from the aforementioned work, Araújo et al. [21] provided the construction for the maximal violation of the odd cycle generalisation of the well-known Klyachko–Can–Binicioğlu–Shumovsky (KCBS) inequality for qutrits [22,23] and even cycle generalisation of Clauser–Horne–Shimony–Holt (CHSH) inequality for two-qubits [24]. Note that the even cycle generalisation of CHSH inequality is similar to Braunstein–Caves inequalities [25], which have been heavily investigated in the literature. For the four cycle case, a simple extension of these even cycle non-contextuality inequalities to the phase space case was provided by Arora et al. [26].

It is important to observe the connections among KS box, non-contextuality inequalities and PR boxes.

1. The KS boxes were motivated by KCBS non-contextuality inequality [18].
2. The KCBS non-contextuality inequality belongs to the same family of inequalities as the CHSH inequality [9].
3. The maximum value of CHSH inequality for no-signalling theories is provided by PR boxes [16].
4. The PR box can be simulated by KS box for $p=\frac{1}{2}$ [18].

In this paper, we further explore the interconnections among the generalised versions of the aforementioned objects, namely generalised PR boxes, arbitrary dimensional KS boxes and *n*-cycle non-contextuality inequalities. Our work provides the pathway for the study of these generalised contextual and nonlocal resources at their junction.

### Paper Structure

In Section 2, we start with studying KS box for the *n*-dimensional case and provide the optimal classical strategy as well as corresponding success probability for simulating the box using classical resources. Our results provide the minimum gap between the optimal classical strategy and the KS box based strategies for arbitrary *p* and *n*. We observe that the optimal success probability for classical simulation decreases monotonically with the dimension of the KS box.

In Section 3, we study the *n*-cycle contextuality scenario and the corresponding non-contextuality inequalities. We explore the odd cycle generalisation of the well-known Klyachko–Can–Binicioğlu–Shumovsky (KCBS) inequality [22,23] and even cycle generalisation of Clauser–Horne–Shimony–Holt (CHSH) inequality [24]. Following the construction provided by Araújo et al. [21], we discuss the necessary and sufficient condition for the violation of the generalised KCBS inequality in Section 3.4 and necessary condition for the violation of even-cycle generalisation of CHSH inequality in Section 3.4. Furthermore, we provide a simple phase space extension of even cycle generalisation of CHSH inequality by harnessing the techniques provided by Arora et al. [26].

Within no-signalling theories, the maximum violation of CHSH inequality is obtained by Popescu–Rohlrich box, also known as PR box [16]. The PR box and its analogue for even-cycle generalisation of CHSH inequality are the contents of Section 4. In their seminal work [18], Bub et al. studied the simulation of a PR box using KS box. We extend the idea to arbitrary dimensional KS box and PR box. We study the joint probability distribution for the KS box and find the criteria for the violation of even-cycle generalisation of CHSH inequality. Given the even cycle generalisation of CHSH inequality, we provide the bound on *p* (tunable parameter) for the KS box required to saturate classical, quantum and no-signalling bounds.

Finally, we conclude in Section 5. We discuss the implications of our study and some interesting open problems for future work.

## 2. Simulating KS Box

**Figure 1:**
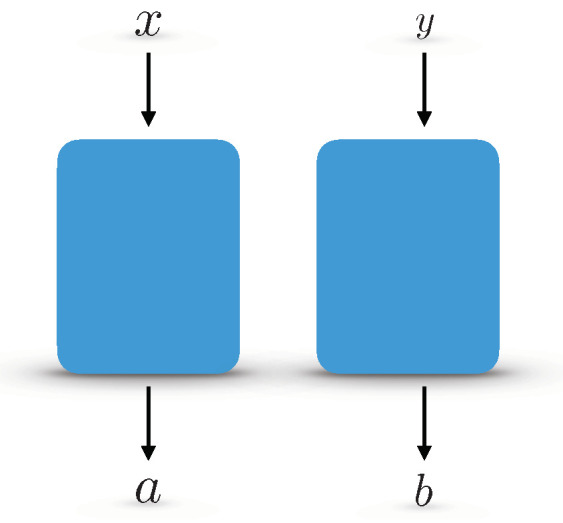
KS box is a bipartite no-signalling box. The value of *a* does not depend on *y* and similarly *b* does not depend on *x*. The box exhibits nonlocal correlations.

A Kochen–Specker–Klyachko box or KS box is a bipartite no-signalling box with two inputs and two outputs (depicted in Figure 1). No-signalling means that the inputs of one sub-part of the box are independent of the output of the complementary part. The outputs are always binary; however, the inputs depend on the dimensionality of the box.

Formally speaking, no-signalling enforces the following constraints:

(1) $$\begin{array}{c} \sum_{b}P\left(a,b|x,y\right)=\sum_{b}P\left(a,b|x,y^{\prime }\right), \end{array}$$

(2) $$\begin{array}{c} \sum_{a}P\left(a,b|x,y\right)=\sum_{a}P\left(a,b|x^{\prime },y\right), \end{array}$$

where P(a,b|x,y) denotes the probability of getting *a* and *b*, when x,y are the input. One can define the box formally as following.

**Definition** **1.** *An N-dimensional Kochen–Specker–Klyachko box or KS box, defined in [18] is a no-signalling resource with two inputs, $x,y\in \left\{1,2,\cdots ,N\right\}$ and two outputs a,b∈{0,1}, which satisfies the following constraints:*

*1.*  *a=b if x=y, and*
*2.*  *a.b=0 if x≠y.*

A KS box with marginal probability *p* for the output “1” is referred to as $KS_{p}$ box. For example, the fraction of “1”s in a $KS_{\frac{1}{5}}$ box is $\frac{1}{5}$. We refer to the KS box condition corresponding to a.b=0 for unequal inputs as ⊥. Given two parties, e.g., Alice and Bob, who are space-like separated, it is not possible to simulate the KS box statistics with full accuracy for arbitrary *p* using classical resources only (for example, some shared randomness) [18]. We want to find the probability of successful simulation of KS box statistics for various strategies. This is an important question because any classical strategy will only produce the best Bell-local statistics and thus the amount by which it fails to simulate a Bell-nonlocal resource such as KS can can be used to quantify the Bell non-locality of KS box.

**Figure 2:**
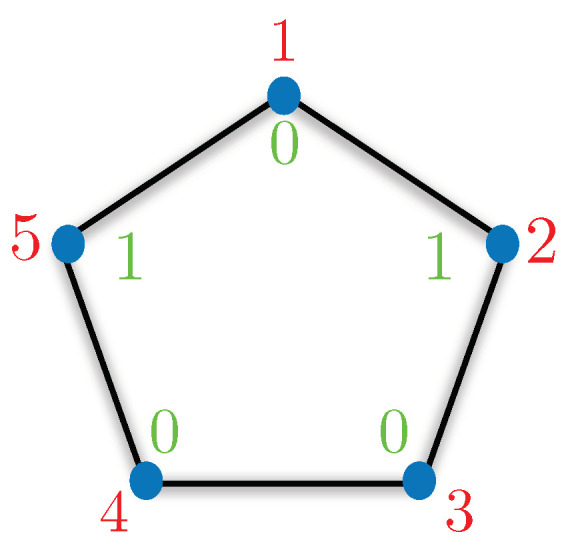
Chart $C_{2}$ for a five-dimensional KS box corresponds to two “1s” and three “0s”. The red entries correspond to inputs and the outputs are in green. The above chart fails to simulate the KS box statistics when the inputs are 2 and 5.

To capture the essence of classical strategies, we use the language from graph theory. Consider an *N*-gon with a 0/1 assignment to its vertices. A 0/1 assignment with *M* “1s” for a given *N*-gon corresponding to an *N*-dimensional KS box is referred to as a chart of degree *M*, in short $C_{M}.$ For example, chart $C_{1}$ for a five-dimensional KS box will assign “1” to one of the vertices and “0” to the rest. Please refer to Figure 2, for a pictorial understanding. To simulate the statistics corresponding to KS box, the spatially separated parties (Alice and Bob) will use their pre-shared strategy. The possible strategies can be captured using the charts discussed before. No-communication is allowed between Alice and Bob once the simulation starts. The only classical resource they share is the access to such charts and some shared randomness to decide which chart to use. The shared randomness determines the fraction of times a particular chart can be used in a strategy. For example, suppose they agree to simulate $P=\frac{1}{3}$ using charts $C_{1}$ and $C_{2}$. Then, they must use chart $C_{1}$ with probability $\frac{1}{3}$ and chart $C_{2}$ with probability $\frac{2}{3}$. This can be achieved by using a biased coin which gives head with probability $\frac{2}{3}$ and tail with probability $\frac{1}{3}$. Using chart $C_{0}$ and $C_{1}$ will always satisfy the ⊥ condition. All other charts will violate the ⊥ condition up to varying proportion.

Simulating a $KS_{p}$ box essentially requires the satisfaction of the ⊥ conditions along with the marginal condition. The use of charts already guarantees equal outputs for same inputs.

**Proposition** **1.** *Given the chart $C_{M}$, the probability of successful simulation of the* ⊥ *condition is given by*

(3) $$P_{⊥}\left(C_{M}\right)=\frac{N^{2}-M^{2}+M}{N^{2}}.$$

**Proof.**  For an *N*-dimensional KS box, the total number of possible input pairs for Alice and Bob are $N^{2}.$ If they use the chart $C_{M}$ to simulate the KS box, then the probability of failure corresponds to the probability of choosing different inputs with output 1. The number of such edges (with ordering) whose vertices correspond to output 1 is M(M−1). Thus, the probability of successful simulation is

(4) $$1-\frac{M(M-1)}{N^{2}}=\frac{N^{2}-M^{2}+M}{N^{2}}.$$

This completes the proof. □

For $p\leq \frac{1}{N},$ Alice and Bob can use chart $C_{0}$ and $C_{1}$ to simulate the KS box. However, we observe that, to satisfy the marginal constraints for $p>\frac{1}{N},$ one needs to use charts of higher degree, which in turn violates the ⊥ conditions. Therefore, perfect classical simulation of the $KS_{p}$ box only exists for $p\leq \frac{1}{N}.$ We now fix a p≤0.5 and compute the optimal classical simulation probability of the $KS_{p}$ box. Now, we present our result concerning the optimal probability of successful simulation for an *N*-dimensional $KS_{p}$ box for arbitrary *p*.

**Theorem** **1.** *For a given p≤0.5, the charts $C_{M-1}$ and $C_{M}$ (only chart $C_{M}$ in case Np is an integer) are optimal for simulating N-dimensional $KS_{p}$ box, where M=[Np]$\left(\mathrm{ceiling}\;\mathrm{integral}\;\mathrm{value}\right)$ and the optimal probability of simulation is given by*

$$P_{optimal}\left(M,N,p\right)=1-\frac{\left(2Np-M)(M-1\right)}{N^{2}}.$$

**Proof.**  Assume that Alice and Bob play the charts $C_{i}$ with probability $p_{i},$ for $i\in Z^{\geq },$ i.e., the set of non-negative integers. For a given probability distribution $\left\{p_{i}\right\}$ over charts, the probability of successful simulation of $KS_{p}$ box is given by $\sum_{i}p_{i}P_{⊥}\left(C_{i}\right).$ Hence, the optimal simulation probability is given by the following linear program:

$$\begin{array}{cc} \max_{\left\{p_{i}\right\}}\sum_{i}p_{i}P_{⊥}\left(C_{i}\right) & \left(\mathrm{success}\;\mathrm{probability}\right)\\ s.t\sum_{i}p_{i}i=Np & \left(\mathrm{mean}\;\mathrm{condition}\right)\\ \sum_{i}p_{i}=1,p_{i}\geq 0\;\forall i & \left(\mathrm{valid}\;\mathrm{probability}\right) \end{array}$$

Now, observe that the objective function is

$$\begin{array}{cc} \sum_{i}p_{i}P_{⊥}\left(C_{i}\right) & =\frac{1}{N^{2}}\sum_{i}p_{i}\left(N^{2}-i^{2}+i\right)\\ & =1+\frac{p}{N}-\frac{1}{N^{2}}\sum_{i}p_{i}i^{2}, \end{array}$$

where in the second equality we used the mean condition along with the valid probability condition. Hence, maximising the objective function corresponds to minimising the variance term with respect to the probability distribution $\{p_{i}\}.$ The optimisation problem of minimising the variance of a random variable defined on a set of non-negative integral points, over all possible probability distributions, for a fixed given mean, has support size at most two. A simple proof for this is given in Appendix A Proposition A1. Specifically, if the mean (Np) is an integer (e.g., =M), the least variance solution will be $p_{M}=1$ and $p_{i}=0,\forall i\neq M.$ For the case when the mean is not an integer, the least variance solution corresponds to a support containing M−1 and *M*, with M=[Np], which follows from simple convexity arguments. With this support, we can compute $p_{M-1}$ and $p_{M}$ using the mean condition, which evaluates to $p_{M-1}=M-Np$ and $p_{M}=Np-M+1.$ Plugging this into the success probability function gives us the optimal simulation probability of the $KS_{p}$ box

$$P_{optimal}\left(M,N,p\right)=\frac{1}{N^{2}}\left(2Np-2NpM+N^{2}+M^{2}-M\right)=1-\frac{\left(2Np-M)(M-1\right)}{N^{2}}$$

This completes the proof. □

**Figure 3:**
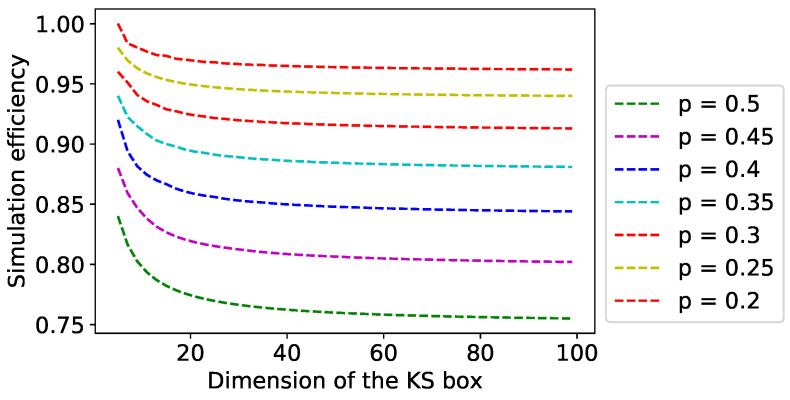
The simulation efficiency has been plotted here as a function of the dimension of the KS box for various marginal probabilities, *p*. It can be seen that the simulation efficiency decreases with dimension for a particular *p*.

Let us have a look at the simulation efficiency in a bit detail. Numerical evidence (refer to Figure 3) suggests that the simulation efficiency decreases with the dimension of the KS box. Please refer to Table 1 for the specific case of p=0.4.

For a particular value of *p*, the nonlocal nature of KS box increases with dimension of the box and hence the simulation efficiency for the optimal classical strategy decreases. Moreover, for a KS box with fix dimension, its nonlocal nature increases with increase in *p*.

Having studied the KS box, we move next to the *n*-cycle non-contextuality inequalities.

## 3. Analysing *n*-Cycle Non-Contextuality Inequalities

Before we analyse the *n*-cycle generalisation of KCBS and CHSH inequalities, we would like to discuss the prior art briefly required to understand our work.

### 3.1. KCBS Inequality

The observables in quantum mechanics are represented as Hermitian matrices. Unlike real or complex numbers, matrices do not commute in general. More importantly, it is possible to have three observables *A*, *B* and *C* such that [A,B]=0, [A,C]=0 but [B,C]≠0. The maximal set of commuting observables defines a context. In the previous example, the observable *A* lies in two contexts defined by the sets {A,B} and {A,C}. Since the observables in a context commute among themselves, they can be measured simultaneously. Given a theory, if the value of an observable in the experiment depends on the context in which it has been measured, the theory is called contextual, otherwise non-contextual. Quantum mechanics is a contextual theory [10]. The experimental tests which can be used to probe the contextual nature of a theory are referred to as contextuality tests. These tests can be often written in terms of an algebraic inequality whose violation witnesses contextuality of the underlying theory. Klyachko–Can–Binicioğlu–Shumovsky (KCBS) inequality is one of the extensively studied state-dependent non-contextuality inequality [8,22,23]. The violation of KCBS inequality by any probabilistic theory rules out its possible completion by a non-contextual hidden variable model. To understand this and the KCBS inequality, let us have a look at the following algebraic quantity (related to KCBS inequality):

(5) $$K=A_{1}A_{2}+A_{2}A_{3}+A_{3}A_{4}+A_{4}A_{5}+A_{5}A_{1},$$

where all the $A_{i}$ can be either +1 or −1. Now, in any theory, where the values of all the $A_{i}$ are predetermined (such is the case in a non-contextual hidden variable theory i.e., the probability theories which can have a non-contextual completion), the average value of Equation (5) is lower bounded by −3. Formally, the KCBS non-contextuality inequality is given by

(6) 〈K〉≥−3,

where 〈K〉 refers to expectation value of *K*. Now, it is possible to have a theory which violates the bound in Equation (6). For example, quantum theory achieves up-to $5-4\sqrt{5}$, which is approximately −3.94427 and hence less than −3. This proves that quantum mechanics is a contextual theory [22]. The measurement setting and the state corresponding to optimal violation of KCBS inequality withIn quantum theory is given by

(7) $$\begin{array}{c} A_{i}=2|v_{i}\rangle \langle v_{i}|-I, \end{array}$$

(8) $$\begin{array}{c} |v_{i}\rangle ={\left(\sin\left(\theta \right)\cos\left(\frac{4\pi i}{5}\right),\sin\left(\theta \right)\sin\left(\frac{4\pi i}{5}\right),\cos\left(\theta \right)\right)}^{T}, \end{array}$$

(9) $$\begin{array}{c} |\psi \rangle ={\left(0,0,1\right)}^{T}, \end{array}$$

where I refers to identity, i∈1,2,3,4,5 and $\cos^{2}\left(\theta \right)=\frac{\cos\left(\frac{\pi }{5}\right)}{1+\cos\left(\frac{\pi }{5}\right)}$. By doing a basis transformation, one can view the KCBS inequality as a state-dependent non-contextuality inequality with five dichotomic measurements with 0/1 outcome. Explicitly,

$$P_{i}=\frac{1+A_{i}}{2},$$

transforms $A_{i}$ with ±1 outcome space to $P_{i}$ with 0/1 outcome space such that

- $P_{i}$ and $P_{i}+1$ are compatible and
- $P_{i}$ and $P_{i+1}$ are exclusive.

Here, addition is taken modulo 5 and exclusivity means that $P_{i}$ and $P_{i+1}$ cannot have outcome 1. This exclusivity corresponding to projective measurements and their outcomes can be captured using a graph, known as “exclusivity graph”. The exclusivity graph approach to contextuality has been studied extensively in the literature and it is important to review the basics of this framework [8]. The nodes of an exclusivity graph correspond to event where an event is constituted by the combination of measurement and corresponding outcome. For example, $\left(a|i\right)$ is an event which corresponds to getting outcome “*a*” for measurement “*i*”. Let us represent the probability of getting outcome “1” given the input was “*i*” as $P\left(1|i\right)$. The events follow exclusivity relation according to the exclusivity graph (a pentagon in the case of KCBS). The exclusivity relation induces following constraint:

(10) $$P\left(1|i\right)+P\left(1|j\right)\leq 1,$$

**Figure 4:**
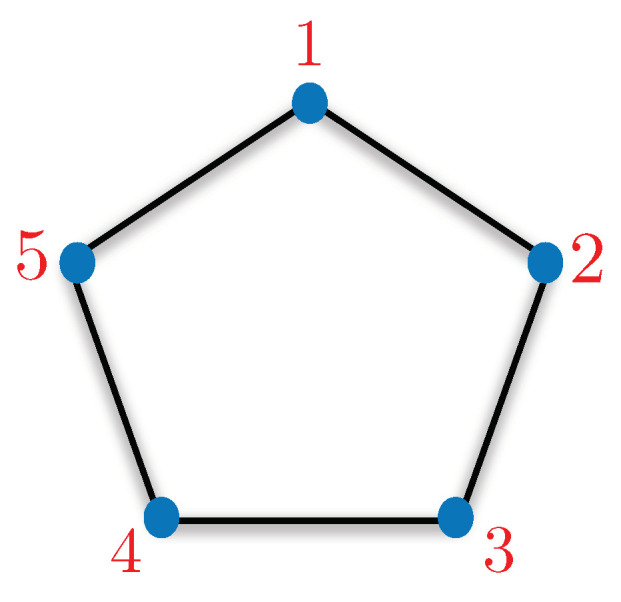
The exclusivity graph corresponding to the KCBS inequality is a pentagon. The inequality involves five events of type $\left(1|i\right)$ where i∈{1,2,3,4,5}. The bound on the inequality for non-contextual hidden variable theories is 2. Quantum theory achieves up to $\sqrt{5}$ and thus manifests the contextual nature of quantum theory.

∀i,j∈E, where *E* corresponds to the edge set of the exclusivity graph. The KCBS inequality corresponds to sum of probabilities assigned to five events of the kind $\left(1|i\right)$ with exclusivity relation following a pentagon (refer to Figure 4).

Given a non-contextuality inequality, the upper bound for non-contextual hidden variable (NCHV) theories is given by independence number of the underlying exclusivity graph, denoted by α(G) [8]. The upper bound for quantum theories is given by Lovász theta number, represented as ϑ(G) [8]. Formally, in graph theoretic language, the KCBS inequality is given by

(11) $$\sum_{i=1}^{5}P\left(1|i\right)\leq \alpha \left(C_{5}\right),$$

where $C_{5}$ represents pentagon and $\alpha (C_{5})$ is equal to 2. The quantum bound corresponds to $\vartheta (C_{5})$ and is equal to $\sqrt{5}$. Since $\vartheta \left(C_{5}\right)>\alpha \left(C_{5}\right),$ it witnesses the contextual nature of quantum theory [8,22].

### 3.2. CHSH Inequality

CHSH inequality is a special case of non-contextuality inequality where the context is provided by space-like separation of parties involved, e.g., Alice and Bob. The scenario corresponding to inequality corresponds to four measurements, two for each party. Each of Alice’s measurements are compatible with Bob’s measurements and vice versa. Suppose Alice’s measurements are given by $A_{1},A_{2}$ and Bob’s measurements are given by $B_{1},B_{2}$. The outcomes corresponding to the measurements are either +1 or −1. The CHSH inequality is given by

(12) $$\langle C_{4}\rangle =\langle A_{1}B_{1}\rangle +\langle A_{1}B_{2}\rangle +\langle A_{2}B_{1}\rangle -\langle A_{2}B_{2}\rangle \leq 2.$$

The local hidden variable theories respect the bound in (12), however quantum theory achieves up-to $2\sqrt{2}$ with appropriate measurement settings and state [24]. These optimal measurement settings and state corresponding to maximal quantum violation are given by,

(13) $$\begin{array}{c} A_{1}=Z⊗I,\;\;A_{2}=X⊗I, \end{array}$$

(14) $$\begin{array}{c} B_{1}=I⊗\frac{-Z-X}{\sqrt{2}},\;\;B_{2}=I⊗\frac{Z-X}{\sqrt{2}}, \end{array}$$

(15) $$\begin{array}{c} |\psi \rangle =\frac{|01\rangle -|10\rangle }{\sqrt{2}}, \end{array}$$

where *X* and *Z* are Pauli matrices, I is identity and |ψ〉 is a Bell state.

### 3.3. Analysing the Generalised KCBS Inequality

The inequality in Equation (11) has been further extended to general odd cycle, which is

(16) $$\sum_{i=1}^{n}P\left(1|i\right)\leq \frac{n-1}{2}.$$

The odd cycle generalisation of KCBS inequality has been studied extensively in literature [13,21,23,27]. Surprisingly, $\frac{n-1}{2}$ corresponds to independence number of the graph for odd cycle case [8,23,28]. The maximum quantum violation for generalised KCBS inequality corresponds to Lovász theta number (denoted by $\vartheta \left(G\right)$), which is $\frac{n\cos\left(\frac{\pi }{n}\right)}{1+\cos\left(\frac{\pi }{n}\right)}.$

We represent the density matrices in the standard basis {|i〉} with matrix elements given by $\rho_{ij}=\langle i|\rho |j\rangle .$ For the odd *n*-cycle generalisation of KCBS inequality, the projectors corresponding to the optimal quantum violation are given by

$$\Pi_{j}=|\psi_{j}\rangle \langle \psi_{j}|$$

where

$$|\psi_{j}\rangle ={\left(\sin\left(\theta \right)\cos\left(\frac{j\pi \left(n-1\right)}{n}\right),\sin\left(\theta \right)\sin\left(\frac{j\pi \left(n-1\right)}{n}\right),\cos\left(\theta \right)\right)}^{T}$$

and $\cos^{2}\left(\theta \right)=\frac{\cos\left(\frac{\pi }{n}\right)}{1+\cos\left(\frac{\pi }{n}\right)}.$ Now, we present the condition under which a qutrit will violate the generalised KCBS inequality for the above measurement settings.

**Proposition** **2.** *A qutrit violates the odd n-cycle generalisation of KCBS non-contextuality inequality if and only if $\rho_{33}\geq \left(\frac{\cos\left(\frac{\pi }{n}\right)\left(n-1\right)-1}{n\left(2\cos\left(\frac{\pi }{n}\right)-1\right)}\right)$.*

**Proof.**  The generalised KCBS operator for the odd *n*-cycle scenario can be defined as

$$K_{n}=\sum_{j=1}^{n}\Pi_{j}.$$

Adding all the projectors ($\Pi_{j}$s), we get

$$K_{n}=\sum_{i=1}^{3}k_{i}|\phi_{i}\rangle \langle \phi_{i}|$$

where

$$|\phi_{1}\rangle =\left(\begin{array}{c} 1\\ 0\\ 0 \end{array}\right),|\phi_{2}\rangle =\left(\begin{array}{c} 0\\ 1\\ 0 \end{array}\right),|\phi_{3}\rangle =\left(\begin{array}{c} 0\\ 0\\ 1 \end{array}\right)$$

and

$$k_{1}=\frac{1}{1+\cos\left(\frac{\pi }{n}\right)}\sum_{j=1}^{n}\cos^{2}\left(\frac{j\pi \left(n-1\right)}{n}\right)$$

$$k_{2}=\frac{1}{1+\cos\left(\frac{\pi }{n}\right)}\sum_{j=1}^{n}\sin^{2}\left(\frac{j\pi \left(n-1\right)}{n}\right)$$

$$k_{3}=n\cos^{2}\left(\theta \right)=\frac{n\cos\left(\frac{\pi }{n}\right)}{1+\cos\left(\frac{\pi }{n}\right)}.$$

Since $\sum_{j}\cos^{2}\left(\frac{j\pi \left(n-1\right)}{n}\right)=\sum_{j}\sin^{2}\left(\frac{j\pi \left(n-1\right)}{n}\right)=\frac{n}{2},$ we get

$$k_{1}=k_{2}=\frac{n}{2\left(1+\cos\left(\frac{\pi }{n}\right)\right)}$$

$$k_{3}=\frac{n\cos\left(\frac{\pi }{n}\right)}{1+\cos\left(\frac{\pi }{n}\right)}$$

The odd *n*-cycle non-contextuality inequality is written as

$$\langle K_{n}\rangle \leq \frac{n-1}{2},$$

where $\langle K_{n}\rangle$ corresponds to the expectation value of the generalised KCBS operator with respect to the underlying preparation. In terms of quantum expectation, the inequality is given by

$$\mathrm{Tr}\left(K_{n}\rho \right)\leq \frac{n-1}{2}.$$

Note that the generalised KCBS operator is diagonal in standard basis and leads to the following simplification:

$$\frac{n}{2\left(1+\cos\left(\frac{\pi }{n}\right)\right)}\left[\rho_{11}+\rho_{22}\right]+\frac{n\cos\left(\frac{\pi }{n}\right)}{1+\cos\left(\frac{\pi }{n}\right)}\left[\rho_{33}\right]\leq \frac{n-1}{2}.$$

Since the trace of a density matrix is always 1, the condition for the violation of odd *n*-cycle non-contextuality inequality becomes;

$$\rho_{33}>\frac{\left(n-1\right)\left(1+\cos\left(\frac{\pi }{n}\right)\right)-n}{n\left(2\cos\left(\frac{\pi }{n}\right)-1\right)}.$$

Simplifying the above expression, we get

(17) $$\rho_{33}>\frac{\cos\left(\frac{\pi }{n}\right)\left(n-1\right)-1}{n\left(2\cos\left(\frac{\pi }{n}\right)-1\right)}.$$

This completes the proof. □

**Figure 5:**
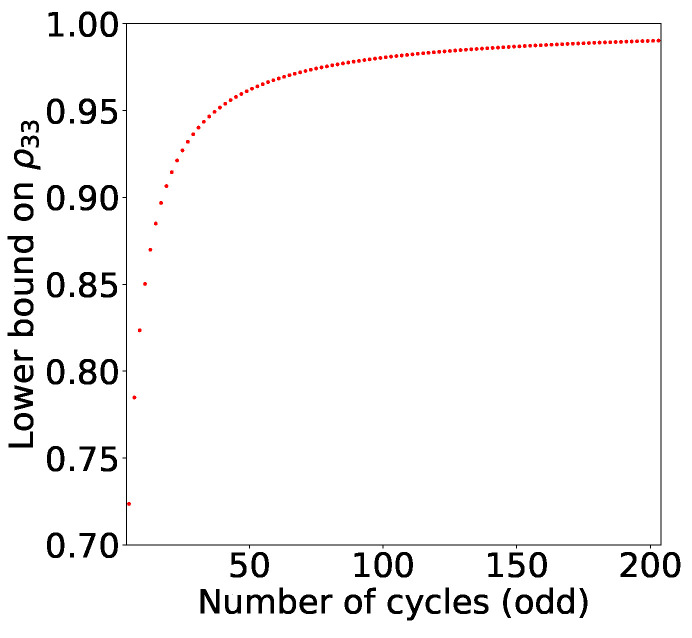
The condition for the quantum violation of the odd *n*-cycle generalisation of KCBS inequality is computed. Lower bound on $\rho_{33}$ for odd *n*-cycle graph has been plotted as a function of n. The set of states which can violate the KCBS inequality corresponding to optimal measurement setting shrinks as we increase n.

**Remark** **1.** *We can see that the set of quantum states for qutrits, which can violate odd n-cycle non-contextuality inequality, shrinks as we increase n (See Figure 5). In the infinite n scenario, the only qutrit which violates the inequality is the pure state $|\psi \rangle ={\left(0,0,1\right)}^{T}$!*

### 3.4. Analysing Chained Bell Inequalities

The *n*-cycle generalisation of CHSH inequality is referred to as chained Bell inequality [21,25]. The even *n*-cycle scenario has *n* measurements i.e., $\left\{X_{1},X_{2},\cdots ,X_{n}\right\}$. All of these are dichotomic measurements with possible outcomes ±1. The chained Bell inequality of cycle *n* is given by

(18) $$\sum_{j=1}^{n-1}\langle X_{j}X_{j+1}\rangle -\langle X_{n}X_{1}\rangle \leq n-2.$$

The optimal construction [21] for violation of this inequality corresponds to $X_{j}=\tilde{X_{j}}⊗I$ for even *j* and $X_{j}=I⊗\tilde{X_{j}}$ for odd *j*, where

(19) $$\tilde{X_{j}}=\cos\left(\frac{j\pi }{n}\right)\sigma_{x}+\sin\left(\frac{j\pi }{n}\right)\sigma_{z}.$$

We now provide the necessary condition for the quantum violation of a chained Bell inequality corresponding to optimal quantum measurement settings.

**Proposition** **3.** *For a given two qubit state, the necessary condition for the quantum violation of chained Bell inequality of cycle n is given by the difference of its extremal eigenvalues i.e.,*

(20) $$\lambda_{1}-\lambda_{4}>\frac{n-2}{n}.$$

**Proof.**  For even *j*,

$$X_{j}X_{j+1}=\tilde{X_{j}}⊗\tilde{X_{j+1}}$$

(21) $$=\left[\cos\left(\frac{j\pi }{n}\right)\sigma_{x}+\sin\left(\frac{j\pi }{n}\right)\sigma_{z}\right]⊗\left[\cos\left(\frac{\left(j+1\right)\pi }{n}\right)\sigma_{x}+\sin\left(\frac{\left(j+1\right)\pi }{n}\right)\sigma_{z}\right].$$

Similarly for odd *j*,

(22) $$X_{j}X_{j+1}=\left[\cos\left(\frac{\left(j+1\right)\pi }{n}\right)\sigma_{x}+\sin\left(\frac{\left(j+1\right)\pi }{n}\right)\sigma_{z}\right]⊗\left[\cos\left(\frac{j\pi }{n}\right)\sigma_{x}+\sin\left(\frac{j\pi }{n}\right)\sigma_{z}\right].$$

Further,

$$X_{n}X_{1}=\tilde{X_{n}}⊗\tilde{X_{1}}$$

(23) $$=-\cos\left(\frac{\pi }{n}\right)\sigma_{x}⊗\sigma_{x}-\sin\left(\frac{\pi }{n}\right)\sigma_{x}⊗\sigma_{z}.$$

Using Equations (21)–(23) and basic arithmetics, the *n*-cycle chained Bell inequality for quantum systems transforms as

$$\frac{n}{2}\cos\left(\frac{\pi }{n}\right)\langle \sigma_{x}⊗\sigma_{x}\rangle +\frac{n}{2}\cos\left(\frac{\pi }{n}\right)\langle \sigma_{z}⊗\sigma_{z}\rangle +\frac{n}{2}\sin\left(\frac{\pi }{n}\right)\langle \sigma_{x}⊗\sigma_{z}\rangle -\frac{n}{2}\sin\left(\frac{\pi }{n}\right)\langle \sigma_{z}⊗\sigma_{x}\rangle \leq n-2,$$

which further simplifies to

$$\cos\left(\frac{\pi }{n}\right)\left[\langle \sigma_{x}⊗\sigma_{x}\rangle +\langle \sigma_{z}⊗\sigma_{z}\rangle \right]+\sin\left(\frac{\pi }{n}\right)\left[\langle \sigma_{x}⊗\sigma_{z}\rangle -\langle \sigma_{z}⊗\sigma_{x}\rangle \right]\leq \frac{2\left(n-2\right)}{n}.$$

For a two qubit density matrix ρ, this translates into

(24) $$\mathrm{Tr}\left(O_{n}\rho \right)\leq \frac{2\left(n-2\right)}{n},$$

where $O_{n}=\cos\left(\frac{\pi }{n}\right)\left[\sigma_{x}⊗\sigma_{x}+\sigma_{z}⊗\sigma_{z}\right]+\sin\left(\frac{\pi }{n}\right)\left[\sigma_{x}⊗\sigma_{z}-\sigma_{z}⊗\sigma_{x}\right].$

The condition for violation of *n*-cycle chained Bell inequality becomes

(25) $$\mathrm{Tr}\left(O_{n}\rho \right)>\frac{2\left(n-2\right)}{n}$$

The eigenvalues of $O_{n}$ are 2,0,0,−2. Suppose the eigenvalues of ρ are $\lambda_{1}\geq \lambda_{2}\geq \lambda_{3}\geq \lambda_{4},$ then

(26) $$\mathrm{Tr}\left(O_{n}\rho \right)\leq 2\left(\lambda_{1}-\lambda_{4}\right).$$

Using Equations (26) and (25), the necessary condition for the violation of *n*-cycle chained Bell inequality turns out to be

(27) $$\lambda_{1}-\lambda_{4}>\frac{n-2}{n}.$$

This completes the proof. □

The set of quantum states form a convex set. Since the non-contextuality inequality in Equation (18) is a linear inequality, its maximum over quantum sets is attained at the extreme points i.e., for pure states. Mixed-ness may lead to non-violation of the aforementioned linear inequality. In this light, it is necessary to study the upper bound on $\lambda_{4}$ (if any).

Since the system under consideration is a two qubit density matrix. We have the following constraints on the eigenvalues:

(28) $$0\leq \lambda_{i}\leq 1\;\;\forall i\in \left\{1,2,3,4\right\}$$

and

(29) $$\sum_{i=1}^{4}\lambda_{i}=1.$$

The constraints in Equations (28) and (29) imply that

(30) $$\lambda_{1}+\lambda_{4}\leq 1.$$

Using Equations (20) and (30), we get

(31) $$\lambda_{4}\leq \frac{1}{n}.$$

The Equation (31) provides an upper bound on $\lambda_{4}$.

**Figure 6:**
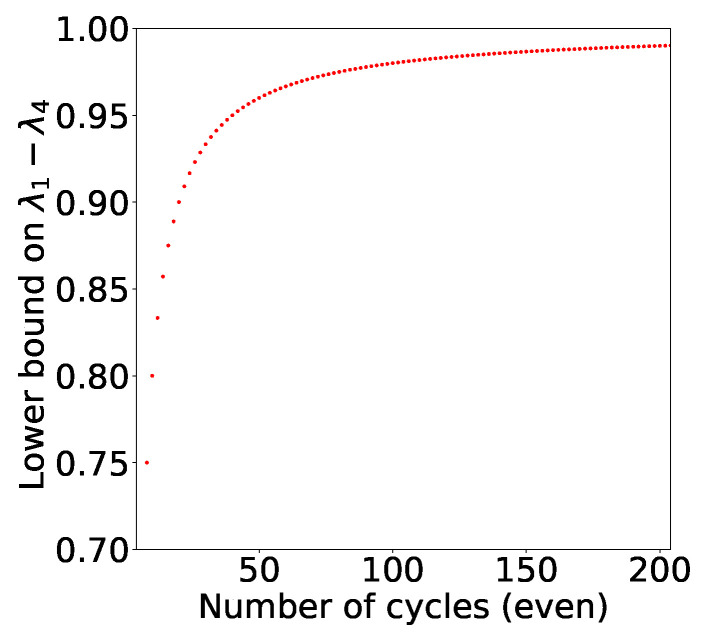
Here, we plot the lower bound on the difference of extremal eigenvalues of a two qubit density matrix as a function of even values of *n*. The set of two qubit quantum states, which could potentially violate chained Bell inequality (as our is necessary and not sufficient), shrinks as we increase n. In the infinite *n* scenario, the only two qubit state that might violate the inequality is Bell state!

**Remark** **2.** *It is easy to see that set of two-qubit quantum states that can violate chained Bell inequality shrinks as we increase n (See Figure 6). In the infinite n scenario, the only two qubit state that violates the inequality is a Bell state!*

The even cycle non-contextuality inequalities can be extended to phase space case quite easily following the work of Arora et al. [26], where the authors provided the phase space extension for n=4. We have already discussed the construction corresponding to the maximal violation of inequality in Equation (18). The inequality is maximally violated by ${\left(0,1/\sqrt{2},-1/\sqrt{2},0\right)}^{T}$ and the maximum violation is $n\cos\left(\pi /n\right).$ We define the following non-contextuality operator in this regard,

(32) $$C_{n}=\sum_{j=1}^{n-1}X_{j}X_{j+1}-X_{n}X_{1}.$$

We know that

(33) $$\exp\left(\iota \theta n.\sigma \right)\sigma \exp\left(-\iota \theta n.\sigma \right)=\sigma \cos\left(2\theta \right)+n\times \sigma \sin\left(2\theta \right)+n\;n.\sigma \left(1-\cos\left(2\theta \right)\right)$$

For $\sigma =\sigma_{x}\hat{x}$ and $n=\hat{z},$

(34) $$\exp\left(\iota \theta \sigma_{z}\right)\sigma_{x}\exp\left(-\iota \theta \sigma_{z}\right)=\sigma_{x}\cos\left(2\theta \right)+\sigma_{y}\sin\left(2\theta \right)$$

Let us look back at the operator in Equation (19) more closely. This can be thought of as $\sigma_{x}$ rotated around *z*-axis with angle $\left(\frac{j\pi }{2n}\right).$ To get the phase space representation, let us start with the quantum mechanical translational operator $\exp\left(\frac{-\iota pL}{\hbar }\right),$ which translates a particle by distance *L*. This operator is not Hermitian and hence we introduce the following symmetric combination to make it Hermitian,

(35) $$X\left(0\right)\equiv \frac{e^{-\iota pL/\hbar }+e^{\iota pL/\hbar }}{2}=\cos\left(\frac{pL}{\hbar }\right).$$

Let $U\left(\phi \right)=\exp\left(\frac{\iota Z\phi }{2}\right)$ where $Z=\mathrm{sgn}\left(\sin\left(\frac{q\pi }{L}\right)\right).$ One can easily see that $X\left(\phi \right)\equiv U^{†}\left(\phi \right)X\left(0\right)U^{†}\left(\phi \right)$ and $\tilde{X_{j}}=X\left(\frac{j\pi }{n}\right).$

Let $\phi \left(q\right)=\langle q|\phi \rangle$ be the localised quantum state symmetric about $q=\frac{L}{2},$ for some length scale *L*. and $\phi_{n}\left(q\right)\equiv \phi \left(q-nL\right).$ Using this construction, the following states are defined:

(36) $$|\psi_{0}\rangle \equiv \frac{1}{\sqrt{M}}\sum_{n=-\frac{M}{2}}^{n=\frac{M-1}{2}}|\phi_{2n+1}\rangle$$

(37) $$|\psi_{1}\rangle \equiv \frac{1}{\sqrt{M}}\sum_{n=-\frac{M}{2}}^{n=\frac{M-1}{2}}|\phi_{2n}\rangle$$

Let $|\psi_{+}\rangle \equiv \frac{|\psi_{0}\rangle +|\psi_{1}\rangle }{\sqrt{2}}$ and $|\psi_{-}\rangle \equiv \frac{|\psi_{0}\rangle -|\psi_{1}\rangle }{\sqrt{2}}.$ Interestingly, for N=2M,

(38) $$\langle \psi_{+}\left|X\right|\psi_{+}\rangle =\left(\frac{N-1}{N}\right),$$

and

(39) $$\langle \psi_{-}\left|X\right|\psi_{-}\rangle =-\left(\frac{N-1}{N}\right).$$

The appropriate entangled state which shows the violation is

(40) $$|\psi \rangle \equiv \frac{|\psi_{+}\rangle_{1}|\psi_{-}\rangle_{2}-|\psi_{-}\rangle_{1}{|\psi_{+}\rangle }_{2}}{\sqrt{2}}.$$

The quantum violation for the state in Equation (40) corresponds to the maximum quantum violation i.e., $n\cos\left(\frac{\pi }{n}\right)$ for large *N*. The experimental implementation of the phase space extension is quite simple and follows directly from the work of Arora et al. [26].

## 4. Simulating PR Box

The KS box is a powerful resource which can be used to efficiently simulate the most non-local no-signalling box, i.e., PR box [16]. The PR box has initially been defined as the box which allows maximum violation of the CHSH inequality in no-signalling theories. One can generalise the notion of PR box corresponding to chained Bell inequalities.

**Definition** **2.** *A PR box is a no-signalling resource with input pair x,y and corresponding output pair a,b where each of these variables takes their values from the set $\left\{0,1\right\}.$ The statistics of the PR box follows the following relation:*

(41) xy=a⊕b,

*which means that the outputs are different if and only if the inputs are x=y=1, otherwise the outputs are same. The PR box can be generalised for input pair $\left(x,y\right)\in {\left\{1,2,\cdots ,n\right\}}^{2}$ and output from the set $\left\{0,1\right\}$ such that outputs are same when inputs are anything except {1,1}. When inputs are {1,1}, the outputs must be different.*

Now, suppose Alice and Bob are equipped with an arbitrary dimensional KS box. Table 2 gives the joint probabilities for an *n*-dimensional $KS_{p}$ box.

KS box is more powerful than PR box and can be used to simulate the same [18]. We ask whether Alice and Bob can simulate a generalised PR box (as defined before) using $KS_{p}$ box. The answer is in the affirmative, and we provide a simple strategy to do so.

**Proposition** **4.** *A PR box of dimension (number of inputs for each party) n can be simulated efficiently using a KS box of dimension 2n−1 with marginal value of $p=\frac{1}{2}$.*

**Proof.**  To prove our claim, we provide the following strategy: Alice relabels her inputs for PR box as follows:

1→1,2→2,3→4,4→6⋯,n→2n−2.

Similarly, Bob relabels his inputs as follows:

1→1,2→3,3→5,4→7⋯,n→2n−1.

The relabelled inputs are used as fresh input for the $KS_{\frac{1}{2}}$ box. Alice outputs what she gets as output from the $KS_{\frac{1}{2}}$. Bob flips his output from $KS_{\frac{1}{2}}$ box in every round and outputs the resultant value. This strategy simulates the statistics corresponding to generalised PR box. □

Given the even cycle generalisation of CHSH inequality, the marginal probabilities *p* in the $KS_{p}$ required to saturate classical bound, quantum bound and no-signalling bound are given by

$$p_{c}\leq \frac{n-2}{2(n-1)},$$

$$p_{q}\leq \frac{n\left(\cos\left(\frac{\pi }{n}\right)+1\right)-2}{4(n-1)}$$

and

(42) $$p_{\mathrm{NS}}\leq \frac{1}{2}$$

respectively.

**Figure 7:**
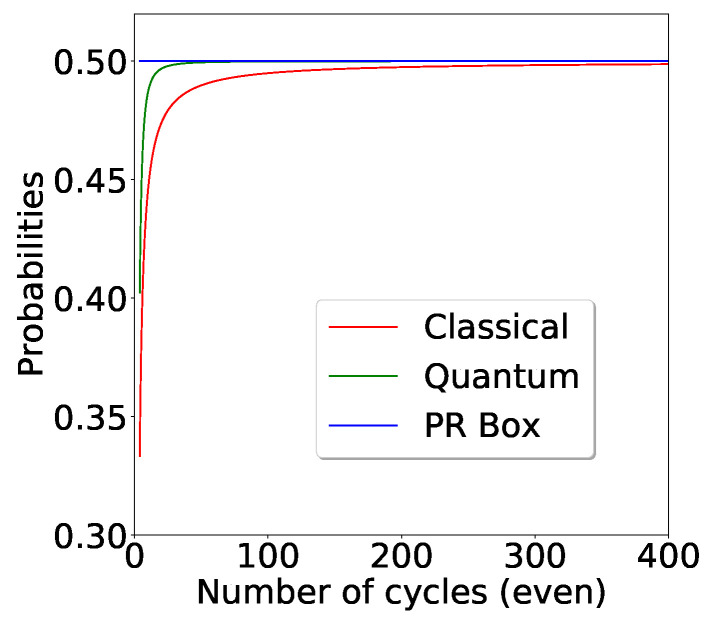
We look at KS box probabilities in various regimes. Note that the quantum probability approaches the PR box limit faster than classical probability as we increase the number of cycles.

**Remark** **3.** *For a large value of n, all the above probability expressions tend to one half. However, the quantum probability approaches the PR box limit of $\frac{1}{2}$ significantly faster than the classical probability.For large n, all these probabilities approach $\frac{1}{2}$ (see Figure 7).*

## 5. Conclusions

We studied arbitrary dimensional KS box, generalised PR box and *n*-cycle non-contextuality inequalities in this work. We provided the optimal classical strategy and the corresponding success probability for classically simulating the KS box. For future work, it is worth exploring the optimal quantum strategy for this purpose. We provided the sufficient condition for the violation of the generalised KCBS inequality and necessary condition for the violation of even-cycle generalisation of CHSH inequality. We also discussed the phase space extension of even-cycle generalisation of CHSH inequality. We leave the phase space extension of KCBS and generalised KCBS inequality for future work. We also studied the strategy for simulating a generalised PR box using KS box. It is also interesting to explore further how the generalised PR box, arbitrary dimensional KS box and *n*-cycle non-contextuality inequalities are related to each other and their implications.

Our work helps quantify the Bell non-locality of KS box in terms of impossibility of classical simulation for general *n*-dimensional case. We also provided the sufficient condition for violation of odd *n*-cycle non-contextuality inequalities. Since contextuality is the chief resource behind various models of quantum computation, our result can help select the resources required to get necessary quantum speed-up. Moreover, our phase space extension of chained Bell inequalities make them suitable for experimental purposes and also harness the underlying Bell non-locality for various quantum communication tasks such as secure key distribution for example. 

## Figures and Tables

**Table 1:** The simulation efficiency decreases with the dimension of the KS box.

Dimension	Marginal Probability	Simulation Efficiency
5	0.4	0.92
7	0.4	0.893878
9	0.4	0.881481
11	0.4	0.87438
13	0.4	0.869822
15	0.4	0.866667
17	0.4	0.862976

**Table 2:** The table displays the joint probabilities for an n-dimensional KS${}_{p}$ box. Note that each of the blocks along the diagonal are same and similarly all the off diagonal blocks are same. Within a block, the top left element is the probability of getting (0,0), top right signifies the probability of getting (0,1), bottom left indicates the corresponding value for (1,0) and, the probability for (1,1) is indicated by the bottom right entry.

	*x*	1		2		⋯		*n*		
y										
1		1−p	0	1−2p	*p*	⋱		1−2p	*p*	
		0	*p*	*p*	0			*p*	0	
2		1−2p	*p*	1−p	0	⋱		1−2p	*p*	
		*p*	0	0	*p*			*p*	0	
⋮		⋱		⋱		⋱		⋱		
*n*		1−2p	*p*	1−2p	*p*	⋱		1−p	0	
		*p*	0	*p*	0			0	*p*	

## Appendix A

**Proposition** **A1.**
*The optimisation problem of minimising the variance of a random variable defined on a set of non-negative integral points, over all possible probability distributions, for a fixed given mean, say =m has support size at most two.*

**Proof.** We make use of the Karush–Kuhn–Tucker (KKT) conditions for deriving necessary optimality conditions. KKT conditions ensure that the gradient of the objective function is perpendicular to the constrained set and the constraints are satisfied. Note that these KKT conditions are just extensions of the Lagrange multiplier method, where now we also have inequality constraints. The Lagrangian for the above optimisation problem is given by

$$L\left(p,\lambda ,\mu ,\nu \right)=\sum_{i}i^{2}p_{i}-\lambda \left(\sum_{i}p_{i}-1\right)+\mu \left(\sum_{i}ip_{i}-m\right)-\sum_{i}\nu_{i}p_{i},$$

where λ and $\nu_{i}\geq 0\;\mathrm{for}\;\mathrm{all}\;i,$ are the KKT multiplier corresponding to the valid probability constraint, and μ is the KKT multiplier corresponding to the fixed mean condition. A necessary condition for optimality is derived by taking the partial derivative of the Lagrangian with respect to $p_{i}$ and setting it to zero, thus getting $i^{2}-\lambda +\mu i=\nu_{i}$. Another necessary KKT condition for optimality is the complementary slackness, which implies $\nu_{i}p_{i}=0$, for all *i*. These two conditions give us $p_{i}\left(i^{2}-\lambda +\mu i\right)=0$, for all *i*. The term inside the brackets is a quadratic expression in terms of *i*, implying that it can be equal to zero for at most two distinct values of *i*. This, in turn, tells us that $p_{i}=0$, for at least all but two *i*, or, in other words, *p* has support size at most 2. □
